# Clinical experience with planning, quality assurance, and delivery of burst‐mode modulated arc therapy

**DOI:** 10.1120/jacmp.v17i5.6253

**Published:** 2016-09-08

**Authors:** Kristofer Kainz, Douglas Prah, Ergun Ahunbay, X. Allen Li

**Affiliations:** ^1^ Department of Radiation Oncology Medical College of Wisconsin Milwaukee WI USA

**Keywords:** IMRT, VMAT, Monte Carlo, low‐dose‐rate radiotherapy, flattening filter‐free beams

## Abstract

“Burst‐mode” modulated arc therapy (hereafter referred to as “mARC”) is a form of volumetric‐modulated arc therapy characterized by variable gantry rotation speed, static MLCs while the radiation beam is on, and MLC repositioning while the beam is off. We present our clinical experience with the planning techniques and plan quality assurance measurements of mARC delivery. Clinical mARC plans for five representative cases (prostate, low‐dose‐rate brain, brain with partial‐arc vertex fields, pancreas, and liver SBRT) were generated using a Monte Carlo–based treatment planning system. A conventional‐dose‐rate flat 6 MV and a high‐dose‐rate non‐flat 7 MV beam are available for planning and delivery. mARC plans for intact‐prostate cases can typically be created using one 360° arc, and treatment times per fraction seldom exceed 6 min using the flat beam; using the nonflat beam results in slightly higher MU per fraction, but also in delivery times less than 4 min and with reduced mean dose to distal organs at risk. mARC also has utility in low‐dose‐rate brain irradiation; mARC fields can be designed which deliver a uniform 20 cGy dose to the PTV in approximately 3‐minute intervals, making it a viable alternative to conventional 3D CRT. For brain cases using noncoplanar arcs, delivery time is approximately six min using the nonflat beam. For pancreas cases using the nonflat beam, two overlapping 360° arcs are required, and delivery times are approximately 10 min. For liver SBRT, the time to deliver 800 cGy per fraction is at least 12 min. Plan QA measurements indicate that the mARC delivery is consistent with the plan calculation for all cases. mARC has been incorporated into routine practice within our clinic; currently, on average approximately 15 patients per day are treated using mARC; and with the exception of LDR brain cases, all are treated using the nonflat beam.

PACS number(s): 87.55.D‐, 87.55.K‐, 87.53.Ay. 87.56.N‐

## I. INTRODUCTION

A novel rotational IMRT delivery technique, referred to throughout this report as “mARC,” has been developed by Siemens Oncology Care Systems and has recently been released for clinical use. mARC applies a “burst‐mode” delivery technique,^(1)^ preliminary studies of which have been reported by Salter et al.^(2)^ for nonflat 7 MV photon beams and by Kainz et al.^(3)^ for conventionally flattened 6 MV photon beams. Burst‐mode delivery is conceptually similar to previously developed arc therapy techniques such as helical tomotherapy,^(4)^ multiple‐arc intensity‐modulated arc therapy (IMAT),^(5)^ and single‐arc volumetric‐modulated arc therapy (VMAT).^6^, ^7^ The distinguishing characteristic of the mARC burst‐mode delivery is that the beam is off when the MLC leaves are in transition between each optimization point (OP), and the angular velocity of the gantry rotation is variable within each OP. The beam is turned on during a small gantry‐angle interval (“arclet”) straddling the OP. When the linac is equipped with a high‐dose‐rate beam, mARC is similar to step‐and‐shoot delivery; the planning and verification of such an arc therapy treatment is simplified.

Although mARC is conceptually similar to helical tomotherapy and VMAT, it offers some advantages over the latter two techniques. First, during an mARC delivery the beam is turned off while the MLCs are in motion. This leads to less blocking of the beam than either tomotherapy or VMAT; this may explain why a comparison of mARC and VMAT plans demonstrated that mARC resulted in fewer monitor units per fraction than did VMAT.^(3)^ Second, mARC delivery more closely resembles the static‐beam approximations made within the planning system, which makes delivery verification with mARC more intuitive compared to dynamic‐MLC techniques such as VMAT. A comparison of mARC, tomotherapy, and VMAT plans demonstrated that all three techniques provided comparable PTV dose coverage, although the PTV uniformity was best with tomotherapy. With regard to organ‐at‐risk (OAR) avoidance, in general all three techniques were comparable.^(3)^

Clinical treatments using mARC have been available in the United States since 2013;^(8)^ our institution has been using mARC clinically since January of 2014. In this report, we present our recent experience in the planning techniques, quality assurance (QA) measurements, and delivery of mARC plans that were generated using a Monte Carlo‐based planning system with a two‐step optimization algorithm, applying both flattened and unflat beams.

## II. MATERIALS AND METHODS

Two different beam modalities have been commissioned for mARC delivery at our institution. One is the traditional 6 MV beam with a flattening filter (FF) in place. This beam, hereafter referred to as “6MV FF,” has a nominal dose rate of 300 MU/min, and is calibrated to deliver 1 cGy per MU at a depth of 1.6 cm at isocenter for a field size of 10cm±10cm and a source‐to‐axis distance (SAD) of 100 cm. The other beam commissioned for mARC is a flattening filter‐free (FFF) beam with a nominal accelerating potential of 7 MV. This beam, hereafter referred to as “7MV FFF,” has a nominal dose rate of 2,000 MU/min, and is calibrated to deliver 1 cGy per MU at a depth of 1.9 cm at isocenter for a field size of 10cm±10cm and 100 cm SAD. At our institution, two Siemens Artiste linear accelerators (Siemens Medical Solutions USA, Malvern, PA) are equipped with mARC delivery capability along with the 6MV FF and 7MV FFF modalities.

All clinical mARC plans delivered at our institution were created using the Monaco treatment planning system version 5.00 (Elekta, Inc., Crawley, UK). Both the 6MV FF and 7MV FFF beams were commissioned for use by the MC (Monte Carlo) v1.6 algorithm. In practice, the collimator angle is set to 10° or 350°, to avoid the definition of purely collinear beams that may make optimization difficult. If during planning it is determined that multiple arcs for a given plane of rotation are required, this is achieved by explicitly creating two or more arcs for the same plane; e.g., a clockwise arc followed by a counterclockwise arc. For plans using the 7MV FFF beam, a minimum of 4 MU per arclet is forced within Monaco, in order to avoid possible problems with nonlinearity for small MU per segment. For 6MV FF mARC plans, arclets with MU as low as 1 MU are permissible. With the exception of brain and head‐and‐neck plans, the Artiste couch geometry and composition was included in the Monaco plan to account for couch attenuation.

Upon approval of each mARC treatment plan, but before patient treatment, two QA procedures are performed upon the plan. A plan delivery QA procedure is performed using the ArcCHECK 2.5‐D diode array (Sun Nuclear Corporation, Melbourne, FL), a cylindrical detector which consists of 1,386 diodes arranged in a helical pattern just beneath the surface of the cylinder. Absolute dose measurements were performed by calibrating a subset of the diode array within a static 10cm±10cm 6 MV beam; no supplementary ionization chambers were inserted within the center of the ArcCHECK detector. The measurement was considered to be consistent with the planning system calculation if the two data sets agreed to within 3% in absolute dose and within 3 mm distance‐to‐agreement for 95% or more of the diodes receiving at least 5 cGy. A secondary MU check is also performed using the RadCalc version 6.2 program (LifeLine Software Inc., Austin, TX). Provided with each arc field's beam energy and segment shapes along with a reference calculation point, RadCalc estimates the required MU for each arc field using Clarkson integration. Note that discrepancies between RadCalc and Monaco can arise due to substantial tissue heterogeneity within Monaco (RadCalc assumes uniform tissue density) or to the location of the calculation point in a low‐dose region. The RadCalc calculation supplements, but does not substitute, the plan delivery QA measurement.

The mARC plans are reported for five cases, which represent a cross section of the mARC treatments performed at our institution. They include: 1) an intact prostate cancer case, 2) a brain case treated using an effectively low dose rate (LDR), 3) another brain case incorporating a partial‐arc vertex field, 4) a pancreatic cancer case, and 5) a liver SBRT case. For each case, we present the prescribed total dose and dose per fraction, the volume of the PTV, the delivery time, and the results of the plan delivery QA measurement (diode pass rate) and RadCalc MU verification calculation.

### A. Case descriptions

#### A.1 Case 1: Intact prostate only

Intact prostate cases are well‐suited for mARC treatments using the 7MV FFF beam, given the relatively central location of the PTV (aligned with the central axis of the nonflat beam profile). This case was clinically treated with a single 360° arc field (as is typical for intact prostate) using the 7MV FFF beam. The prescribed dose was 180 cGy per fraction for 42 fractions, for a cumulative dose of 7,560 cGy to the $112\;{\text{cm}}^{3}$ PTV. To determine whether using a nonflat beam instead of a flat beam was advantageous for mARC prostate treatment, comparative 6MV FF mARC plans were created for six intact prostate cases (including the Case 1 described in this section) that were clinically planned and treated using the 7MV FFF beam. The 6MV FF mARC plans were derived using the same planning CT image set, structure set, prescribed dose, dose per fraction, and set of optimization parameters as were used for the clinically administered 7MV FFF plans; the 6MV FF beam model was the only difference.

#### A.2 Case 2: Low‐dose‐rate brain

For certain histologies of the central nervous system, external beam therapy may be administered at low dose rates (LDR) in an attempt to exploit an enhanced radiosensitivity of tumor cells below a threshold dose rate.^9^, ^10^, ^11^ Tomé and Howard^(12)^ found, in an investigation of glioma cell lines, that administering 20 cGy within 3‐minute intervals corresponded to a dose rate below the early checkpoint. This 20 cGy per 3‐minute interval LDR delivery scheme has been applied clinically at our institution, primarily using conventional 3D CRT planning with static gantry fields and physical wedges. If the total prescribed dose per fraction is 200 cGy, then 10 fields are planned and delivered in 3‐minute intervals: the first field is initiated at 0 min, the second field at 3 min, the third field at 6 min, and so on. For 3D CRT LDR delivery, the therapists must use a stopwatch to indicate when to initiate each field, as neither the linac control console nor the verify‐and‐record system can automate this procedure.

Intensity‐modulated arc therapy is a desirable alternative to 3D CRT for LDR irradiation, offering the advantages of a uniform dose to the PTV per 20 cGy field and improved PTV conformity; Tyagi et al.^(13)^ have reported on VMAT planning and delivery of LDR brain cases using the Pinnacle^3^ treatment planning system (Philips Radiation Oncology Systems, Fitchburg, WI) and Elekta linear accelerators (Elekta, Inc., Crawley, UK). At our institution to‐date, mARC has been used to administer a low‐dose‐rate (LDR) treatment to 17 patients. For our institution's LDR brain cases, the planning technique is as follows. The prescribed dose is typically 200 cGy per fraction, and the cumulative dose is typically 5,400 cGy (as it was for Case 2). A Monaco mARC plan is generated so that a single 360° arc would administer 20 cGy to the PTV; this arc is repeated 10 times, every 3 min, to administer the full 200 cGy per fraction. Although the cumulative prescribed dose is 5,400 cGy, Monaco does not permit the user to set the number of fractions to 270. In order to obtain a proper 5,400 cGy cumulative dose distribution, first a scaled‐down Monaco plan is optimized to provide a cumulative dose of 540 cGy in 27 fractions (20 cGy per fraction). The resulting arc field is exported to MOSAIQ (IMPAC Medical Systems, Sunnyvale, CA), and duplicated to yield 10 identical arc fields to be administered during each fraction; one of those fields was administered for the plan delivery QA measurement. Then, within Monaco, the planned dose is scaled up to 5,400 cGy, in order to obtain the cumulative isodose and DVHs for the full treatment course.

#### A.3 Case 3: Brain PTV with vertex arc fields

Case 3 is an example of a brain (GBM) treatment using arcs within two planes of rotation, one axial plane (with a couch angle of zero) with two overlapping 360° arcs, and the other delivering a vertex‐like field (with a couch angle of 270° IEC) comprised of two overlapping 180° arcs.

#### A.4 Case 4: Pancreas

For pancreas treatments using mARC, two overlapping 360° arcs are often used because the PTV is somewhat larger than is the case for intact prostate treatments; for example, for the pancreas Case 4, the PTV is 395 cm^3^; achieving uniform dose coverage with the 7MV FFF beam (used for Case 4) with a single arc is difficult. For this plan, the collimator angles for the two arcs were set to 10° and 350°; each arc was comprised of 82 arclets, which is greater than the number of arclets per arc typically required for most intact prostate plans (see Table 1). The prescribed dose was 180 cGy per fraction for 28 fractions, yielding a cumulative dose to the PTV of 5,040 cGy.

**Table 1:** Comparison of 6MV FF and 7MV FFF mARC plans for six intact prostate cases, with regard to total number of segments (arclets), total MU per fraction, and total treatment delivery time in secs.

			*# Segments Total*		*Total MU*		*Total Treatment Time (sec)*	
*Prostate Case*	*PTV (cm^3^)*	*# Arcs*	*6MV FF*	*7MV FFF*	*6MV FF*	*7MV FFF*	*6MV FF*	*7MV FFF*
A	137	2	170	140	686.2	748.3	623	468
B	61	1	49	49	485.1	496.9	274	165
C	63	1	81	81	503.4	507.2	333	244
D	77	^1^	49	49	362.0	351.5	234	145
E (Case 1)	112	^1^	31	31	463.6	494.2	234	117
F	79	1	57	57	524.7	575.2	294	194

#### A.5 Case 5: Liver SBRT

mARC was also applied at our institution toward a stereotactic body radiation therapy (SBRT) treatment of a $110\;{\text{cm}}^{3}$ PTV located within the right inferior region of the liver. The prescribed dose to the PTV was 800 cGy per fraction for 5 fractions, leading to a cumulative dose of 4,000 cGy. Liver SBRT planning guidelines were adapted from Andolino et al.,^(14)^ Tse et al. (2008),^(15)^ and Rule et al. (2011).^(16)^

## III. RESULTS AND DISCUSSION

For each of the five cases in this study, the ArcCHECK‐based mARC plan delivery QA measurement was such that over 95% of the detector elements within the treatment field measured absolute doses consistent with the Monaco plan calculation to within 3% at a location within 3 mm of the measurement point; the pass rates for all plans are summarized in Table 2. Also documented in Table 2 are the results of the RadCalc‐based MU verification calculations for each individual arc of each plan. For cases in which the isodose distribution is relatively uniform, the RadCalc MU calculation agrees with the Monaco MU calculation to within 10%.

**Table 2:** Summary of ArcCHECK plan delivery QA diode pass rates and RadCalc MU verification consistency with Monaco, for each case.

*Case Number*	*Disease Site*	*Arccheck Diode Pass Rate (%)*	*Radcalc MU Verification Consistency with Monaco (%)*
1	prostate	98.5	4.4
2	low‐dose‐rate brain	98.3	−2.8
			1.1
3	brain with vertex arc	95.6	0.3
			0.4
			1.6
4	pancreas	98.5	6.1
			13.8
			1.1
5	liver SBRT	99.6	2.9
			5.8
			5.9

### A.1 Case 1: Intact prostate only

**Figure 1:**
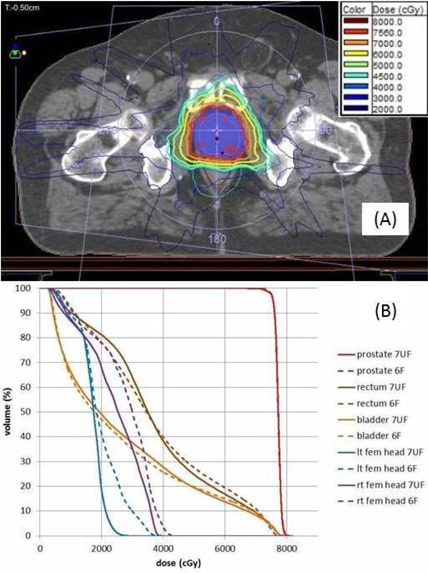
Intact prostate (Case 1): (a) isodose distribution for the mARC 7MV FFF treatment plan, PTV in blue color‐wash, 7560‐cGy isodose line in solid red; (b) dose‐volume histograms for the clinically administered 7MV FFF mARC plan (solid lines) along with a 6MV FF mARC plan generated for comparison (dashed lines).

**Figure 2:**
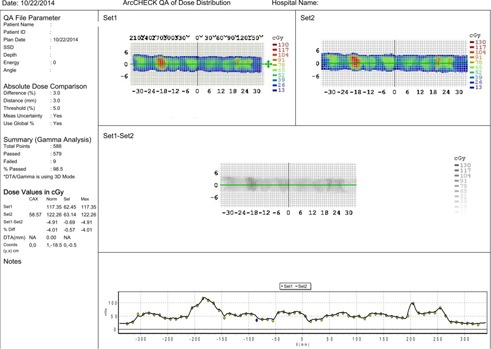
An example of the ArcCHECK plan delivery QA measurement report for Case 1. The upper‐left frame is the measured dose distribution; the upper‐right frame is the calculated dose distribution from the Monaco planning system; and the bottom frame compares the two datasets (points represent diode measurements; line represents Monaco calculation) among the diodes in the midaxial plane of the detector.

The delivery time per fraction was slightly less than 2 min. Figure 1(a) shows the planned isodose distribution in the axial plane corresponding to the Artiste isocenter, which was placed interior to the prostate PTV. Figure 1(b) shows the dose‐volume histogram for the clinically administered 7MV FFF mARC plan (solid lines). For the ArcCHECK plan QA measurement (shown in Fig. 2), the diode pass rate was 98.5% for this plan.

Figure 1(b) includes the DVHs for the mARC plan for Case 1 using the 6MV FF beam model. Although the PTV coverage is nearly identical for the two beam models, the 7MV FFF model resulted in lower dose to both femoral heads. Among the six prostate cases, the FFF plan resulted in lower mean dose to the right femoral head for five cases, and lower mean dose to the left femoral head for four cases. This is expected due to the lateral falloff of the nonflat beam in the vicinity of the femoral heads.

Table 1 summarizes the target volume, number of mARC fields, total number of segments (arclets) among the arcs, total number of MU per fraction, and total fraction delivery time as measured on the Artiste accelerator that had both 6MV FF and 7MV FFF modalities available. For all but the two‐arc Case A, the final Monaco plan resulted in an identical number of segments. For all but one case, the total number of MU per fraction was slightly lower when the 6MV FF model was used. All of our 7MV FFF plans had substantially lower delivery times than did the 6MV FF plans, ranging from 25% to 50% among the cases. This is attributed to the FFF beam's higher dose rate, given that both models have a nearly equal number of segments.

The total number of MU per fraction among our Monaco prostate mARC plans ranged from approximately 350 to approximately 750. Among the Monaco single‐arc prostate plans, our results compare favorably with the results of Eclipse (Varian Medical Systems, Inc., Palo Alto,

CA) mARC plans for 180 cGy per fraction prostate cases using the 7MV FFF beam; Sarkar et al.^(8)^ reported the total MU ranging from approximately 480 to 1,240. Our results are also consistent with those reported using the Prowess Panther (Prowess Inc., Concord, CA) treatment planning system; Dzierma et al.^(17)^ achieved Prowess‐generated mARC prostate plans with MU per fraction ranging from 365 to 452 using 6MV FF photons and 366 to 507 using 7MV FFF beams. One possible explanation for the difference in MU per fraction among single‐arc Monaco, Prowess, and Eclipse plans is that the number of arclets per arcs among our Monaco cases may have been consistent with the Prowess plans in the Dzierma study^(17)^ and considerably less than those for the Eclipse plans in the Sarkar study;^(8)^ unfortunately, neither report summarized the number of arclets per arc among their final plans.

### A.2 Case 2: Low‐dose‐rate brain

**Figure 3:**
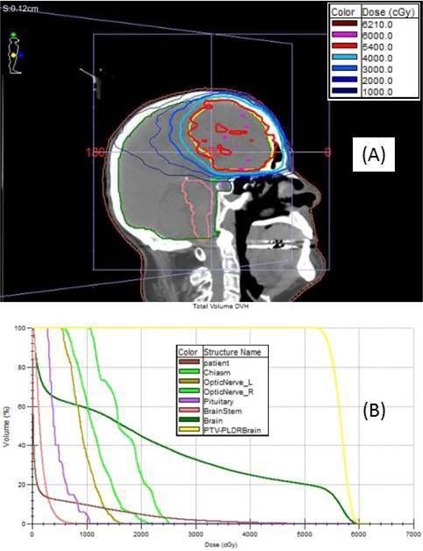
LDR brain (Case 2): (a) axial view of the isodose distribution for the mARC 6MV FF treatment plan — both the PTV (volume 271 cm^3^) and the 5400 cGy isodose line as red lines; (b) DVHs for the 54Gy PTV and for key organs at risk.

For the full treatment course of the LDR brain case, the cumulative isodose distribution is shown in Fig. 3(a), and the DVHs are shown in Fig. 3(b). Prior to the first fraction, the time to deliver a single 20 cGy arc was measured, along with the time to return the gantry to the starting point to deliver the next arc. By happenstance, the delivery time and return time for a single arc is approximately 3 min for the 6MV FF beam, 20 cGy delivery, and 300 MU/min rate. For this case, the time to administer a single 20 cGy arc was 2 min 53 sec; with a 1 minute 3 sec time required to return the gantry to the staring position for the next arc, the time between consecutive 20 cGy fields was 3 min 56 secs, which is above the minimum 3‐minute interval required for 20 cGy delivery. Thus, the therapists did not need to monitor a stopwatch during treatment; they needed only to autosequence the 10 fields and administer them in succession; this eliminated missing the 3‐minute interval as a potential source of treatment error. Also, unlike 3D CRT LDR treatments, for mARC LDR treatments it was not necessary to explicitly enter a reduced MU/min rate into the verify‐and‐record system or the control console. The ArcCHECK plan delivery QA indicated a pass rate of 98.3%, and the RadCalc MU verification calculation was 2.8% below the Monaco calculation.

To date, only the 6MV FF beam has been used for LDR brain treatments. The 7MV FFF might be considered if a 20 cGy arc using the 6MV FF model takes too long; however, the restriction of ≥4MU per segment might make it difficult to create a 7MV FFF mARC LDR plan that would pass the QA measurement.

### A.3 Case 3: Brain PTV with vertex arc fields

**Figure 4:**
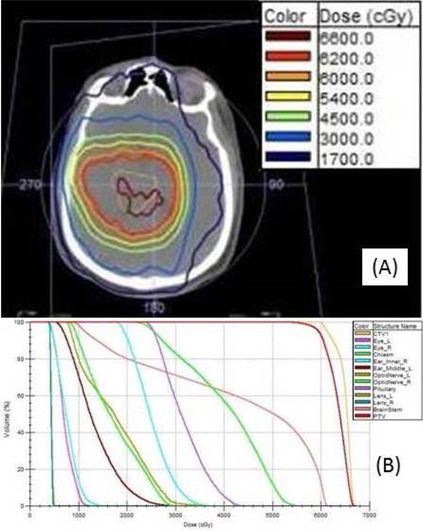
Brain (Case 3): (a) isodose distribution for the mARC 7MV FFF treatment plan, PTV (volume 205 cm^3^) as a thin red line, 6000 cGy isodose line in thick orange; (b) the plan DVHs, including the PTV in red. Note that the maximum doses to both lenses are approximately 500 cGy.

Figure 4(a) shows the planned isodose distribution, and Fig. 4(b) shows the plan DVH. The prescribed dose was 200 cGy per fraction in 30 fractions for a cumulative dose to the PTV of 6,000 cGy. The 7MV FFF beam model was used; the resulting delivery time for all four mARC fields per fraction was 6 min and 11 secs.

**Figure 5:**
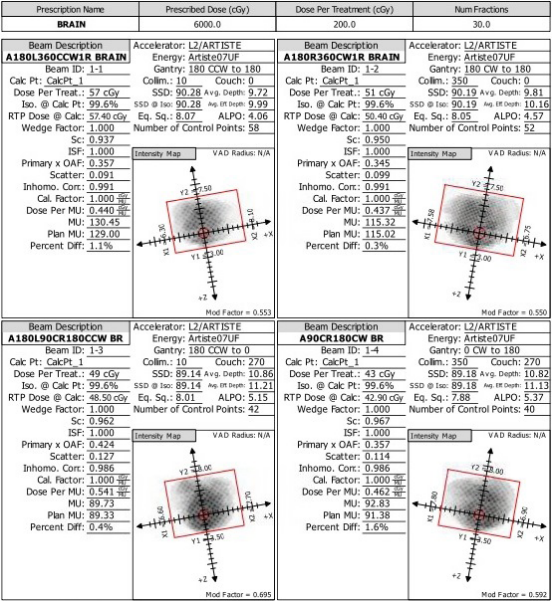
Brain (Case 3): results of the RadCalc MU verification calculations for each of the four mARC fields.

For arc therapy plans incorporating a nonzero couch angle, the QA plan is generated in Monaco with the couch angle for all beams set to zero, so as to avoid directly irradiating the ArcCHECK electronics during the QA measurement. The pass rate was 95.6%. A RadCalc MU verification was performed for this case; a view of the RadCalc report is shown in Fig. 5. Among all beams, the RadCalc MU calculation was within 1.6% of the Monaco calculation, likely because the PTV is in a relatively homogeneous medium and the calculation point is in a region of high and uniform dose.

### A.4 Case 4: Pancreas

**Figure 6:**
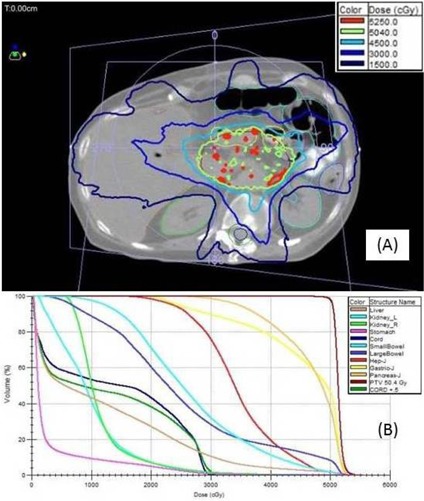
Pancreas (Case 4): (a) isodose distribution for the mARC 7MV FFF treatment plan, PTV (volume 395 cm^3^) a thin red line, the 5040 cGy isodose line light green; (b) the plan DVHs, including the PTV in dark red.

For the pancreas mARC plan, the isodose distribution is shown in Fig. 6(a), and the DVHs are shown in Fig. 6(b). The ArcCHECK plan delivery QA measurement indicated a pass rate of 98.5% among the diodes in the direct beam. The overall time to deliver both mARC fields was approximately 10 min, which is typical for mARC pancreas treatments using 7MV FFF beams. A RadCalc MU verification calculation was also performed; however, the results were 6.1% and 13.8% different from the Monaco calculations for both beams. This might be due in part to heterogeneities (e.g., bowel gas) apparent in the original planning CT (see Fig. 6(a)).

### A.5 Case 5: Liver SBRT

**Figure 7:**
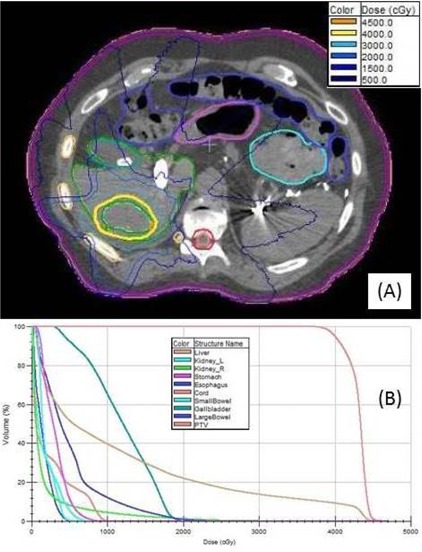
Liver SBRT (Case 5): (a) isodose distribution for the mARC 7MV FFF treatment plan, PTV (volume 110 cm^3^) as a thin green line, the 4000 cGy isodose line in yellow; (b) DVH plots for the liver SBRT PTV and relevant organs at risk from the Monaco plan.

The isodose distributions for the SBRT liver case are shown in Fig. 7(a), and the DVHs are shown in Fig. 7(b). The mARC plan used the 7MV FFF beam model and four 360° arc fields, each with 81 arclets; the cumulative MU for each fraction was 3,304. The full‐fraction delivery time during the plan quality assurance measurement was approximately 12 min; the pass rate among the ArcCHECK diodes was 99.6%. During the patient treatment the arcs were administered within a time frame of 21 min.

## V. CONCLUSIONS

mARC has been incorporated into routine use within our clinic since January of 2014. With two Siemens Artiste linear accelerators with mARC and 7MV FFF capability, our institution treats approximately 15 patients per day using mARC. With the exception of the LDR brain cases, all of these treatments use the 7MV FFF beam.

## COPYRIGHT

This work is licensed under a Creative Commons Attribution 3.0 Unported License.

## Supporting information

Supplementary Material

Supplementary Material

## References

[1] Stahl J and Shukla H , Assignee Siemens AG . System and method for dynamic strobe arc therapy. European Patent EP 2 266 664 A1. December 29, 2010.

[2] Salter BJ , Sarkar V , Wang B , Shukla H , Szegedi M , Rassiah‐Szegedi P . Rotational IMRT delivery using a digital linear accelerator in very high dose rate ‘burst mode’. Phys Med Biol. 2011;56(7):1931–46. 21364260 10.1088/0031-9155/56/7/002

[3] Kainz K , Chen GP , Chang YW , et al. A planning and delivery study of a rotational IMRT technique with burst delivery. Med Phys. 2011;38(9):5104–18. 21978056 10.1118/1.3622612

[4] Mackie TR , Holmes T , Swerdloff S , et al. Tomotherapy: a new concept for the delivery of dynamic conformal radiotherapy. Med Phys. 1993;20(6):1709–19. 8309444 10.1118/1.596958

[5] Yu CX . Intensity‐modulated arc therapy with dynamic multileaf collimation: an alternative to tomotherapy. Phys Med Biol. 1995;40(9):1435–49. 8532757 10.1088/0031-9155/40/9/004

[6] Otto K . Volumetric modulated arc therapy: IMRT in a single gantry arc. Med. Phys. 2008;35(1):310–17. 18293586 10.1118/1.2818738

[7] Bzdusek K , Friberger H , Eriksson K , Hårdemark B , Robinson D , Kaus M . Development and evaluation of an efficient approach to volumetric arc therapy planning. Med Phys. 2009;36(6):2328–39. 19610322 10.1118/1.3132234

[8] Sarkar V , Huang L , Rassiah‐Szegedi P , et al. Planning for mARC treatments with the Eclipse treatment planning system. J Appl Clin Med Phys. 2015;16(2):458–64. 26103202 10.1120/jacmp.v16i2.5351 PMC5690068

[9] Marples B and Collis SJ . Low‐dose hyper‐radiosensitivity: past, present, and future. Int J Radiat Oncol Biol Phys. 2008;70(5):1310–18. 18374221 10.1016/j.ijrobp.2007.11.071

[10] Krueger SA , Collis SJ , Joiner MC , Wilson GD , Marples B . Transition in survival from low‐dose hyper‐radio‐sensitivity to increased radioresistance is independent of activation of ATM Ser 1981 activity. Int J Radiat Oncol Biol Phys. 2007;69(4):1262–71. 17967316 10.1016/j.ijrobp.2007.08.012 PMC12161167

[11] Schultz CJ and Geard CR . Radioresponse of human astrocytic tumors across grade as a function of acute and chronic irradiation. Int J Radiat Oncol Biol Phys. 1990;19(6):1397–403. 2262364 10.1016/0360-3016(90)90350-s

[12] Tomé WA and Howard SP . On the possible increase in local tumour control probability for gliomas exhibiting low dose hyper‐radiosensitivity using a pulsed schedule. Br J Radiol. 2007;80(949):32–37. 16945935 10.1259/bjr/15764945

[13] Tyagi N , Yang K , Sandhu R , et al. External beam pulsed low dose radiotherapy using volumetric modulated arc therapy: planning and delivery. Med Phys. 2013;40(1):011704. 23298074 10.1118/1.4769119

[14] Andolino DL , Johnson CS , Maluccio M , et al. Stereotactic body radiotherapy for primary hepatocellular carcinoma. Int J Radiat Oncol Biol Phys. 2011;81(4):e447–53. 21645977 10.1016/j.ijrobp.2011.04.011

[15] Tse RV , Hawkins M , Lockwood G , et al. Phase I study of individualized stereotactic body radiotherapy for hepatocellular carcinoma and intrahepatic cholangiocarcinoma. J Clin Oncol. 2008;26(4):657–64. 18172187 10.1200/JCO.2007.14.3529

[16] Rule W , Timmerman R , Tong L , et al. Phase I dose‐escalation study of stereotactic body radiotherapy in patients with hepatic metastases. Ann Surg Oncol. 2011;18(4):1081–87. 21046264 10.1245/s10434-010-1405-5

[17] Dzierma Y , Bell K , Palm J , Nuesken F , Licht N , Rübe C . mARC vs. IMRT radiotherapy of the prostate with flat and flattening‐filter‐free beam energies. Radiat Oncol. 2014;9:250. 25424536 10.1186/s13014-014-0250-2 PMC4272773

