# Supplementary figures and images for: Clinical experience with planning, quality assurance, and delivery of burst‐mode modulated arc therapy
Source: J Appl Clin Med Phys. 2016 Sep 8;17(5):47–59. doi: 10.1120/jacmp.v17i5.6253 (PMC5874115; doi:10.1120/jacmp.v17i5.6253)

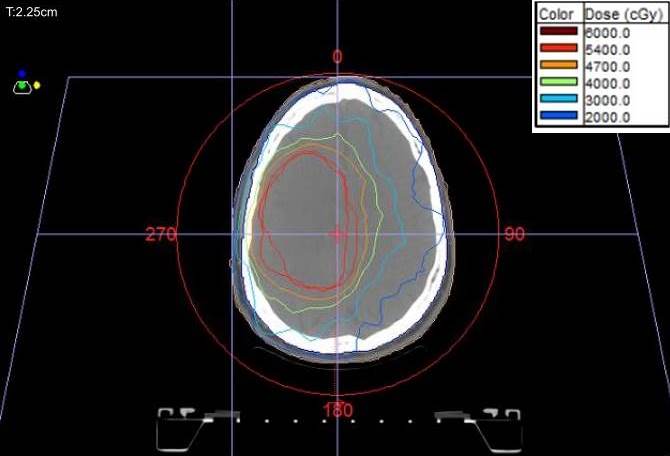

Supplement: Supplementary file 1 — Supplementary Material [file ACM2-17-047-s001.jpg]

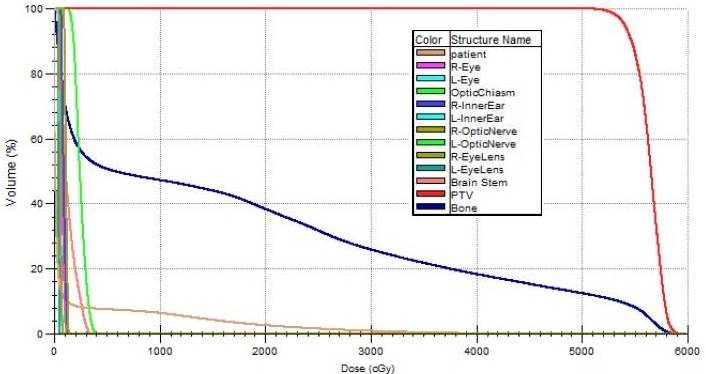

Supplement: Supplementary file 2 — Supplementary Material [file ACM2-17-047-s002.jpg]
